# Renal localization and regulation by dietary phosphate of the MCT14 orphan transporter

**DOI:** 10.1371/journal.pone.0177942

**Published:** 2017-06-29

**Authors:** Thomas Knöpfel, Alexander Atanassoff, Nati Hernando, Jürg Biber, Carsten A. Wagner

**Affiliations:** 1 Institute of Physiology, University of Zurich, Zurich, Switzerland; 2 NCCR Kidney.CH, Switzerland; The University of Manchester, UNITED KINGDOM

## Abstract

MCT14 is an orphan transporter belonging to the SLC16 transporter family mediating the transport of monocarboxylates, aromatic amino acids, creatine, and thyroid hormones. The expression, tissue localization, regulation, and function of MCT14 are unknown. In mouse MCT14 mRNA abundance is highest in kidney. Using a newly developed and validated antibody, MCT14 was localized to the luminal membrane of the thick ascending limb of the loop of Henle colocalizing in the same cells with uromodulin and NKCC2. MCT14 mRNA and protein was found to be highly regulated by dietary phosphate intake in mice being increased by high dietary phosphate intake at both mRNA and protein level. In order to identify the transport substrate(s), we expressed MCT14 in *Xenopus laevis* oocytes where MCT14 was integrated into the plasma membrane. However, no transport was discovered for the classic substrates of the SLC16 family nor for phosphate. In summary, MCT14 is an orphan transporter regulated by phosphate and highly enriched in kidney localizing to the luminal membrane of one specific nephron segment.

## Introduction

Inorganic phosphate is a molecule involved in energy metabolism and signaling as well as essential for structural molecules in cells and bone. Phosphate excess triggers ectopic calcifications and is associated with increased cardiovascular mortality. Therefore, the plasma levels of phosphate are kept in a close range; this is achieved by a variety of compensatory and highly regulated mechanisms. Phosphate deficiency leads to increased levels of plasma active vitamin D, which promotes intestinal phosphate absorption at least in part by stimulating the expression of the Na^+^-dependent phosphate transporter NaPi-IIb [1]. Additionally, hypophosphatemia blunts the production of parathyroid hormone (PTH) [2] and fibroblast growth factor 23 (FGF23) [3], releasing the inhibitory effect of both hormones on renal phosphate cotransporters (NaPi-IIa, NaPi-IIc and PiT-2), thus enhancing renal reabsorption of phosphate. On the other hand hyperphosphatemia leads to an increased renal excretion of phosphate, an effect mediated at least in part by PTH [2] and FGF23 [4,5,6]. Both hormones reduce the renal expression of NaPi-IIa NaPi-IIc and PiT-2 in the proximal tubule [2], and FGF23 additionally suppresses the production of vitamin D[7]. But beyond these effects directly linked to phosphate metabolism, alterations in phosphate intake affect, among other effects, glycogenolysis and glucose production [8], indicating broader systemic changes in response to phosphate status.

The SLC16 gene family comprises 14 members collectively called MCTs (monocarboxylate transporter) due to the fact that the first 4 members to be characterized mediate transport of monocarboxylates such as lactate, pyruvate and ketone bodies, in a proton dependent manner [9]. MCT1, 3 and 4 associate with CD147 (basigin) [10], an immunoglobulin-like protein, that acts as a chaperone for these MCTs. The interaction of MCT1 and CD147 has been extensively studied and it was shown that sorting of MCT1 to the right pole of an epithelial cell relies on CD147 [11]. MCT1 and 2 are both expressed in kidney where they are involved in reabsorbing lactate. MCT1 is localized to the basolateral membrane of the renal proximal tubule together with CD147 [12], whereas MCT2 is found in the basolateral membrane of cells in the thick ascending limb of the loop of Henle [13].

The characterization of further members of the SLC16 family revealed that transport by MCTs is not limited to monocarboxylates. MCT8 transports thyroid hormone [14] and mutations of MCT8 associate with elevated levels of triiodothyronine (T_3_) and psychomotor retardation [15]. MCT10 is an aromatic amino acid transporter, also known as TAT1 (T-type amino acid transporter 1). Both transporters mediate transport in a proton (and sodium) independent manner [16], unlike the first 4 members. Recently MCT12, associated with juvenile cataract, was characterized as a creatine transporter [17], further widening the substrate spectrum of the MCT family. MCT12 localizes to the basolateral membrane of proximal tubules in kidney, and patients with mutated MCT12 excrete increased amounts of guanidinoacetate, but functional studies did not show transport of the creatine precursor [18]. The other members of the family remain orphan transporters, and although it has been suggested that they might act as drug transporters (as demonstrated with bumetanide transport for MCT6 [19]) their endogenous transport substrates are still elusive. A renal transcriptome study detected mRNA of MCT1, MCT2, MCT5, MCT6, MCT7, MCT8, MCT9, MCT10 (TAT1), MCT13 and MCT14 in kidney [20], and the renal localization of MCT1, 2, 7, 8 and 10 has been described [12,21].

MCT14, encoded by the SLC16A14 gene, is an orphan member of the family that has been shown to be expressed in the bovine intestinal tract [22] and the mammary gland of lactating cows [23]. A recent study also mapped the expression of MCT14 in the mouse brain and provided a relative expression of this transporter in several tissues [24]. Of interest, among all the tissues tested renal mRNA abundance was the highest (by about 20 fold). This report also provides a phylogenetic analysis of the MCTs, which shows that MCT14 is closest related to MCT8, 9 and 10 [24], the non-classical members of the family.

Here we report that the renal expression of MCT14 is regulated by the dietary content of phosphate, the generation and validation of a polyclonal antibody to analyze its segmental distribution in kidney as well as our attempts to identify substrates.

## Experimental procedures

### Animals

All experiments with animals were performed according to Swiss Animal Welfare laws and were approved by the local veterinary authority (Veterinäramt Zürich). Male mice (C57BL/6) 10 to 13 weeks old were fed for 14 hours or 5 days with either high (1.2%) or low (0.1%) phosphate diet (Kliba Promivi AG, Switzerland). Food was provided ad libitum and animals had free access to water. After the adaptation, mice were anesthetized with a mixture of ketamine and xylazine. Venous blood was collected from the vena cava and centrifuged (8 min at 8’000rpm at 4°C) in heparinized tubes containing. After centrifugation, the plasma was aliquoted and snap frozen in liquid nitrogen. Organs were extracted and snap frozen. All samples were stored at -80°C until further use.

### RNA isolation and semi-quantitative real-time PCR (RT-qPCR)

Snap frozen kidneys were homogenized in RLT buffer (Qiagen, Germany) containing 1% β-mercaptoethanol. Total RNA was purified from these homogenates using the Qiagen RNeasy Mini Kit following the manufacturer’s protocol. Isolated RNA was reverse transcribed to cDNA with the TaqMan Reverse Transcription Kit (Applied Biosystems), following the manufacturers protocol.

Specific primers for Slc16a14/MCT14 and Cyp27b1 and the corresponding probe labeled with FAM/TAMRA (Mm01272722_m1, Mm01165918_g1; Applied Biosystems) as well as for β-actin (Microsynth, Switzerland) were purchased. TaqMan universal PCR master mix (Applied Biosystems) containing primers and probes in concentrations of 5 μM and 25 μM, respectively, was used to amplify cDNA. Relative mRNA expression levels were measured on a 7500 Fast Real Time PCR System (Applied Biosystems). The cycle number at a given threshold (Ct) was determined, and the relative expression (R) was calculated according to the formula R = 2^((CT– β-actin)–(CT-MCT14), where R represents the expression of MCT14 relative to the housekeeping gene (β-actin).

To analyze the expression of Slc16a14 mRNA in kidneys and oocytes from *Xenopus laevis*, total RNA was prepared and reverse transcribed as indicated above. Then, mRNA, was detected by amplification of cDNA with Slc16a14 specific primers (forward: TGC ATG TGT ACT CCA GCC TG, reverse: TGT CAT AGA TCC AAC CTG CAA, Microsynth, Switzerland) using TaqMan universal PCR master mix (Applied Biosystems), followed by separation on an 1% agarose gel.

### Western blot analysis

Frozen kidneys were homogenized in homogenization buffer (300 mM Mannitol, 5 mM EGTA, 12 mM Tris/HCl, pH 7.1) for 2 min on ice using a Polytron homogenizer (PT 10–35, Kinematica GmbH, Lucerne). The homogenates were then centrifuged for 5 min at 2’000 rpm at 4°C. Supernatants were collected and protein concentrations were determined with the BioRad DC protein assay, based on the Lowry method (44). Aliquots of 50 μg of renal homogenates were mixed with Laemmli sample buffer (0.38 M Tris base, 8% SDS, 4 mM EDTA, 40% (v/v) glycerol, pH 6.8 with HCl, 4 mg/ml of Bromphenol Blue) and loaded on a 10% acrylamide SDS gel for SDS-PAGE. Proteins were transferred from gel onto polyvinylidene difluoride membranes (PVDF; Immobilon-P, Millipore) in a standard tank system (Mini Trans- Blot, Bio-Rad). PVDF membranes were blocked with TBS containing 5% fat-free powder milk at room temperature and incubated with primary antibodies over night at 4°C. Membranes were then washed with TBS and blocked again before incubation with secondary antibodies for 2 hours at room temperature. Horseradish peroxidase-coupled secondary antibodies (GE Healthcare, United Kingdom) were used to detect primary antibody binding. Chemiluminescence was detected upon incubation with HRP substrate (Western Chemiluminescence HRP Substrate, Millipore) for 5 minutes, using a LAS-4000 camera system (Fujifilm).

An antibody targeting a predicted extracellular loop (Uniprot) of MCT14 was raised by immunizing rabbits with an antigenic peptide (NH_2_-KSGGPLGMAEEQDRRPGNEEMVC-COOH) linked to KLH (Pineda, Berlin, Germany). The anti-serum was affinity purified using protein A/G chromatography.

For deglycosylation of renal proteins the PNGase F kit (New England BioLabs inc., Allschwil, Switzerland) was used following the manufacturer’s protocol. Prior to SDS page, renal homogenates were treated with PNGase F under denaturing (0.5% SDS plus 40 mM DTT, at 95°C and room temperature) and non-denaturing (room temperature) conditions. After incubation for 4 hours with PNGase F, samples were mixed with laemmli sample buffer and western blot was performed.

### Immunofluorescence

Animals were perfused through the left cardiac ventricle with a pre-fixative solution (1000 U/ml Heparin, 0.2% procain-HCl, 3.2% CaCl_2_ and 0.18% NaCl) followed by the fixative (3% Paraformaldehyde (PFA)/PBS). After incubation in PFA for one hour, kidneys were placed overnight in 32% sucrose/PBS and subsequently embedded in OCT embedding Matrix (Cell Path, Newtown, Wales, United Kingdom) and frozen in liquid propane. Cryosections of 5 μm were mounted on slides (Superfrost Plus, Thermo Scientific) and blocked with 3% bovine serum albumin/PBS; the primary antibodies against MCT14 (1/1000), NKCC2 [25] (1/1000), Uromodulin [26] (1/400), NCC [27] (1/500) or NaPi-IIa [28] (1/400) were diluted in PBS and added to the cryosections for incubation over night at 4°C. Then, samples were washed twice with hypertonic PBS (18g NaCl/PBS), once with PBS and incubated with the secondary antibodies donkey anti-rabbit Alexa 594, donkey anti-sheep Alexa 488 and goat anti-guinea-pig Alexa 488 (1/1000, Invitrogen) during 2 hours at room temperature. After two consecutive washing steps with hypertonic PBS and once with PBS, cover slips were mounted with Glycergel (DakoCytomation, Baar, Switzerland). Fluorescence was detected with a Leica fluorescence microscope (Leica CTR600). Leica AF lite and Image J freeware programs were used to process the pictures.

### Expression of MCT14 in *Xenopus laevis* oocytes

An *Escherichia coli* clone containing the cDNA of murine Slc16a14 inserted into the pCMV-Sport6 expression vector downstream of the SP6 promotor (IRAVp968E0963D) was purchased from imaGenes (Berlin, Germany). Bacterial clones were selected and expanded using standard procedures. The vector was purified using the QIAprep Spin Miniprep Kit (QIagen), and was linearized with the restriction enzyme NotI. After purification with the QIAquick gel extraction kit (Qiagen), the linearized plasmid served as template to synthesize cRNA using the mMessage mMachine SP6 kit (Ambion) in the presence of the 5’-cap analogue (m7G(5')ppp(5')G (New England BioLabs inc., Allschwil, Switzerland). Upon purification, 10 ng of MCT14 cRNA were injected into oocytes isolated from female *Xenopus laevis*. Three days after cRNA injection, oocytes were used for experiments. Non-injected and water-injected oocytes served as control.

### Oocyte preparation for immunodetection of MCT14

*Xenopus laevis* oocytes were placed in 20 μl lysis buffer (100 mM NaCl, 20 mM Tris HCl (pH 7.6) and 1% Igepal CA 630 (Sigma Aldrich, Buchs, CH)) and lysed by pipetting up and down. Lysates were then centrifuged for 3 minutes at 16000g at 22°C. Yolk-free supernatants were collected avoiding contamination with floating lipids and mixed with Laemmli sample buffer. A volume corresponding to 1/3 of an oocyte/lane was loaded on an acrylamide SDS gel and protein expression was tested following the Western blot protocol described above.

To prepare *Xenopus laevis* oocytes for immunohistological detection, cells were incubated in 3% PFA in PBS for 4 hours at 4°C. After washing with PBS, oocytes were placed in 30% sucrose/PBS over night at 4°C for cryoprotection. Oocytes were then transferred to cryomolds (Cryomold Biopsy, Sakura Finetek Germany GmbH, Staufen, Germany) containing embedding medium (OCT embedding Matrix, Cell Path, Newtown, Wales, United Kingdom) and were immediately frozen in liquid propane. Frozen oocytes were stored at -80°C and processed for immunofluorescence experiments as described above.

### Transport studies in *Xenopus laevis* oocytes expressing MCT14

Ten oocytes were transferred into plastic vials containing ice cold ND96 (96 mM NaCl, 2 mM KCl, 1.8 mM CaCl_2_, 1 mM MgCl_2_, 5 mM Hepes, pH 7.4 adjusted with Tris). ND96 was replaced with ND96 containing 100 μM of the desired uptake substrate together with traces of radioactively labelled substrate (5 μCi/ml). Upon 10 minutes incubation at room temperature, the solution was removed and oocytes were subsequently washed 4 times with ice cold ND96. Single oocytes were transferred to scintillation vials containing 200 μl of 2% SDS and lysed by shaking. Upon addition of Emulsifier-Safe (PerkinElmer), the radioactivity accumulated in each oocyte was measured in a β-counter (Packard BioScience) and total uptake was calculated.

### Statistical analysis

Unpaired students t-test was used to compare expression levels. P-values < 0.05 were considered as significant. Data is presented as Mean + SEM.

## Results and discussion

Phosphate plays an essential role in a large variety of vital functions ranging from cell signaling and energy metabolism to structural purposes. Hence, it is not surprising that changes in phosphate status, as those induced by alterations in dietary phosphate intake, have a broader impact on systemic homeostasis than just adjusting intestinal and renal (re)absorption of phosphate. Thus, dietary alterations in phosphate supply cause a fast response in PTH and later on in vitamin D and FGF23 [4,5], but also promote changes in other metabolic pathways such as gluconeogenesis, or altered insulin sensitivity [8], indicating the importance of phosphate in energy metabolism.

### Dietary phosphate content regulates Slc16a14 mRNA

**Fig 1:**
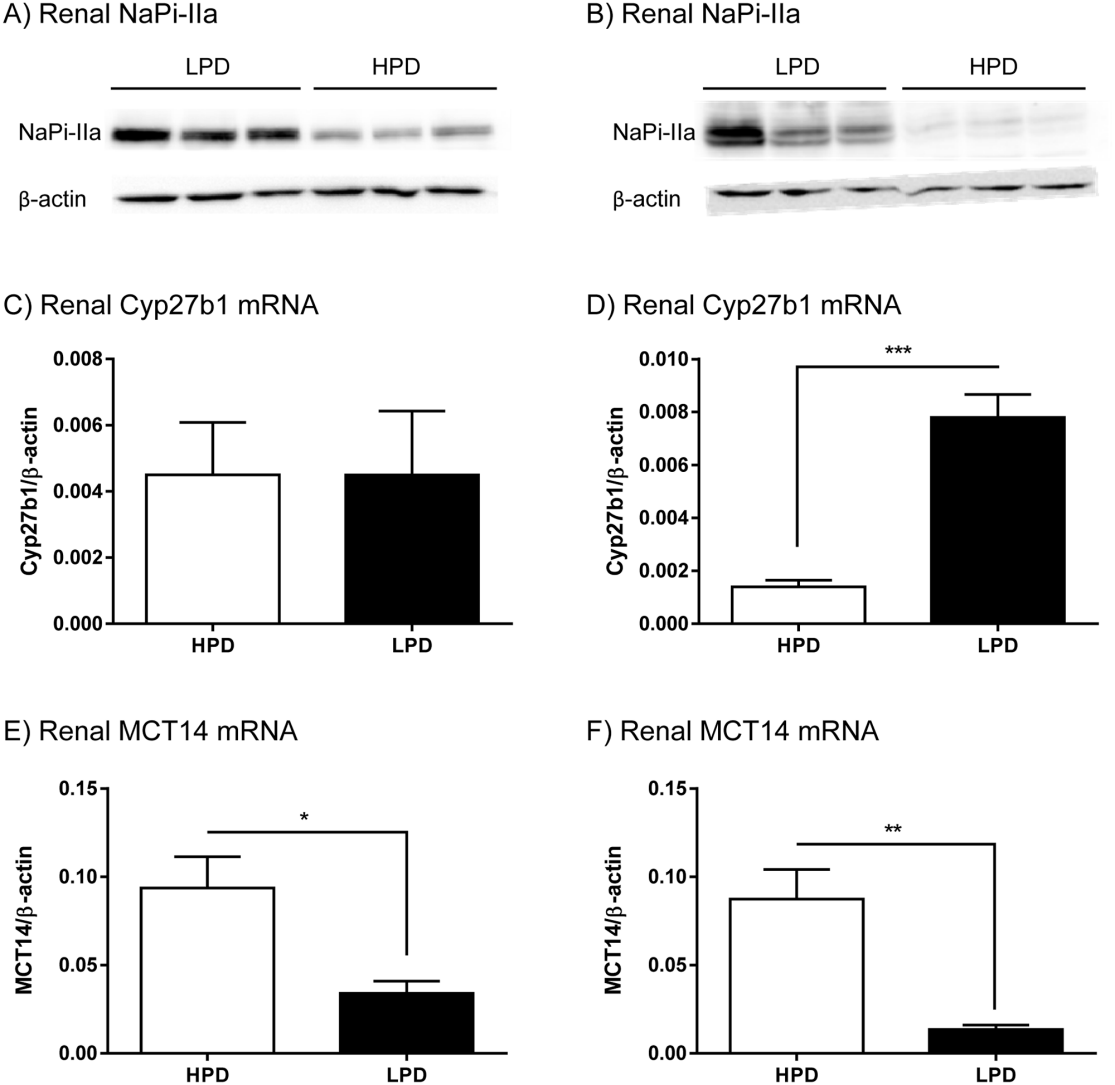
Renal adaptation to low (LPD) and high (HPD) dietary phosphate. Adaptation was confirmed by the expression of NaPi-IIa protein abundance in kidneys of mice fed 14 hours (A) and 5 days (B) with both diets. β-actin was used as a loading control. Renal mRNA expression of Cyp27b1 and MCT14 after either 14 hours (C and E) or 5 days (D and F) of LPD respectively HPD, n = 5–6 animals/group. Statistical differences were calculated with t-test: *p<0.05, **p<0.01 and ***p<0.001.

A transcriptome analysis using GeneChip Mouse Genome 430 2.0 arrays (Affymetrix, Santa Clara, CA) was performed on kidneys from mice adapted to high (1.2% phosphate, HPD) or low (0.1% phosphate, LPD) dietary phosphate for either 14 hours or 5 days, conditions that are considered as acute or chronic adaptation, respectively. Adaptation to the diets was validated by quantifying the renal protein expression of the Na^+^/phosphate cotransporter NaPi-IIa [29]. As previously reported [30], acute (Fig 1A) and chronic (Fig 1B) LPD diets increased the abundance of NaPi-IIa protein compared to HPD. The microarray detected more than 12’000 protein coding transcripts. Only transcripts with at least 1.5 fold change and p-values <0.05 were considered for further analysis. Under LPD conditions 50 transcripts were found to be upregulated acutely and 1259 under chronic adaptation. LPD further decreased the expression of 93 transcripts acutely and 631 chronically (detailed data to be reported elsewhere). To validate the data obtained by microarrays, the mRNA expression of Cyp27b1 (the 1-alpha-hydroxylase responsible for the last step of vitamin D_3_ activation) was independently quantified by RT-PCR. In agreement with previous observations, Cyp27b1 mRNA abundance was not affected by acute changes in dietary phosphate (Fig 1C), whereas chronic adaptation to LDP led to a 10 fold increase (Fig 1D), confirming that vitamin D_3_ is not acutely activated by phosphate restriction [31].

In both, acute and chronic adaptation experiments, transcriptome analysis revealed that the mRNA of Slc16a14 (the gene encoding the monocarboxylate transporter MCT14) was strongly regulated. Among the SLC transporter transcripts, SLC16a14 mRNA appeared to be the most highly affected. In fact Slc16a14 mRNA abundance was decreased by a factor of 4.3 in mice acutely fed a LPD as compared with mice fed a HPD, whereas the reduction was by a factor of 5.0 in chronically adapted mice. The microarray data was confirmed by semi-quantitative real time RT-PCR with renal mRNA extracted from the same group of mice as well as from an independent group of animals. Already 14 hours after the start of the dietary adaptation renal Slc16a14 mRNA expression was drastically decreased in animals under LPD condition compared with HPD (Fig 1E). This effect was further enhanced after chronic adaptation for 5 days (Fig 1F).

### Antibody generation and validation of specificity

**Fig 2:**
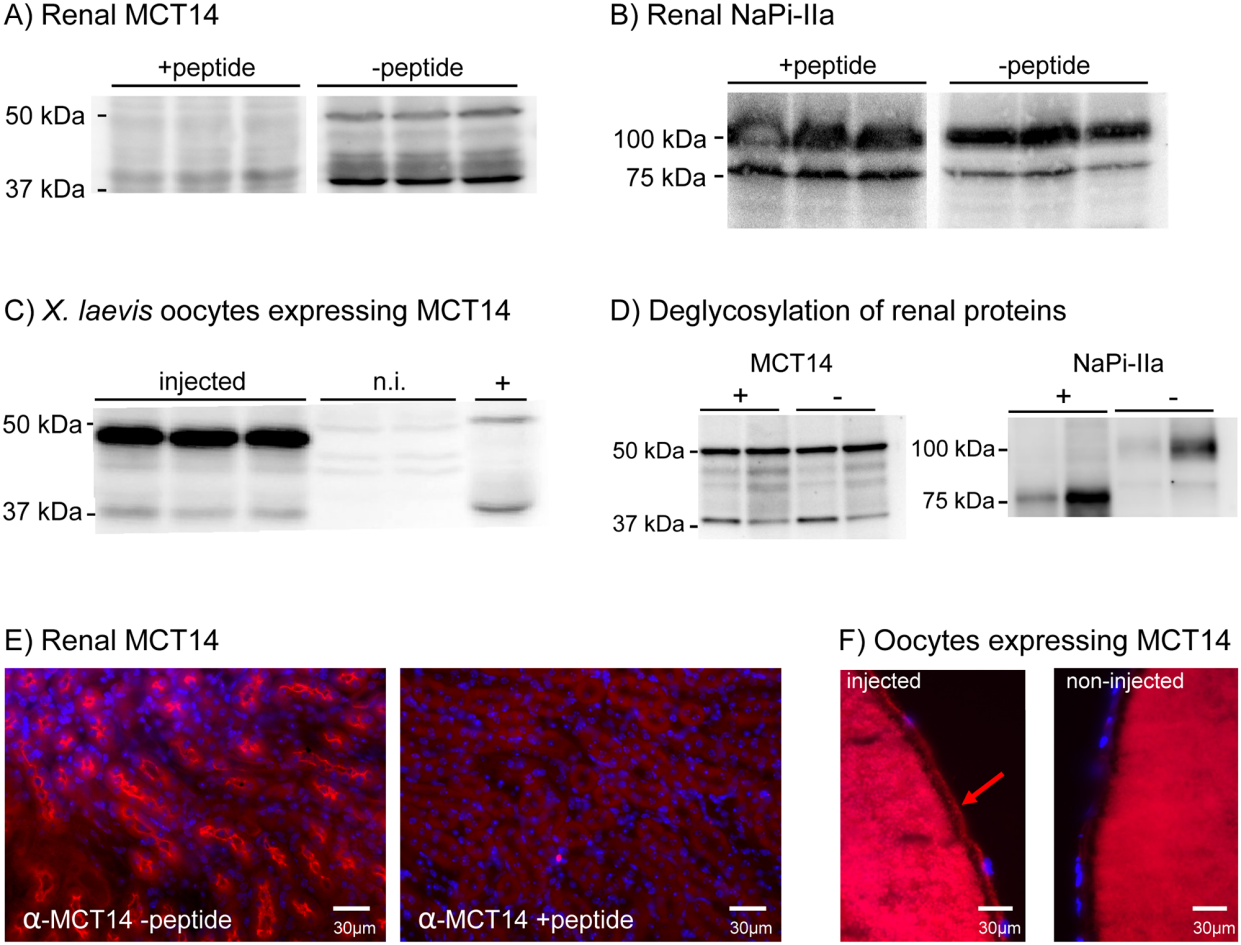
Validation of a newly generated MCT14 antibody. Renal homogenates were incubated with antibodies against MCT14 (A) and NaPi-IIa (B) in the presence and absence of the antigenic peptide as indicated. Lysates of *Xenopus laevis* oocytes injected with MCT14 cRNA and non-injected oocytes (n.i.) were incubated with the MCT14 antibody (C); renal homogenate was loaded in the last lane (+). Deglycosylated (+ PNGase) and native (- PNGase) renal homogenates were incubated with antibodies against MCT14 and NaPi-IIa (D). Immunofluorescence was performed on kidney cryosections in the presence and absence of the antigenic peptide (E). Immunofluorescence in oocytes expressing MCT14 and corresponding non-injected oocytes (F).

Murine MCT14 consists of 510 amino acids and, as for other members of this family, hydropathy plots predict 12 transmembane domains with cytoplasmic N and C-termini [32]. The predicted topology of MCT1 was experimentally confirmed in proteolytic cleavage experiments in red blood cells, where the big intracellular loop and the N- and C-termini were only accessible for cleavage in leaky ghosts [33]. To investigate if the changes in MCT14 mRNA expression were paralleled by altered protein levels, a polyclonal antibody was raised in rabbits against a peptide located in a predicted extracellular loop. Western blot analysis with renal homogenates was performed in the presence and absence of the antigenic peptide to confirm specific binding of the antibody to its epitope (Fig 2A). The anti-MCT14 antibody detected one band of about 50 kDa, which is in good agreement with the expected size of the full length protein, as well as smaller bands at about 40 kDa that may represent either degradation products or unglycosylated proteins, since they were mostly detected in boiled samples. A similar pattern of bands was reported in a recent publication using a commercial antibody [24]. Preincubation with the antigenic peptide abolished detection of both bands but did not affect the binding of the NaPi-IIa [28] antibody in the same homogenates (Fig 2B). Further proof of binding specificity was obtained by western blots of lysates from *Xenopus laevis* oocytes injected with Slc16a14/MCT14 cRNA or left non-injected. In lysates of oocytes injected with Slc16a14/MCT14 cRNA, the antibody detected two bands with molecular sizes of about 40 and 50 kDa corresponding to the major bands protected by the antigenic peptide in renal homogenates. These bands were absent from non-injected oocytes (Fig 2C). To analyze whether the two bands represent different MCT14 glycosylated forms, renal protein samples were treated with PNGase F prior to Western blotting. These deglycosylation experiments did not show any change in the pattern of migration of MCT14 for all the protocols used (Fig 2D), whereas deglycosylation treatment of the same sample decreased the size at which NaPi-IIa was detected (Fig 2D). Hence the two bands observed are rather unlikely to be due to glycosylation.

### Renal localization of MCT14

Real time PCR on mRNA samples extracted from several mouse tissues indicated that the kidney expresses the highest levels of Slc16a14/MCT14 mRNA, followed by pancreas and testis where the mRNA expression levels are about ten times lower than in kidney (data not shown), confirming a previous report [24]. In immunofluorescence experiments on murine kidney cryosections using the newly generated MCT14 antibody, staining was observed mainly in the luminal membrane of nephron segments located in the outer renal medulla and to a lesser extent in the cortex (Fig 2E). Upon co-incubation of the antigenic peptide with the anti-MCT14 antibody, tubular staining was abolished (Fig 2E). These findings indicate specific staining of MCT14 in kidney sections using a standard immunofluorescence protocol.

**Fig 3:**
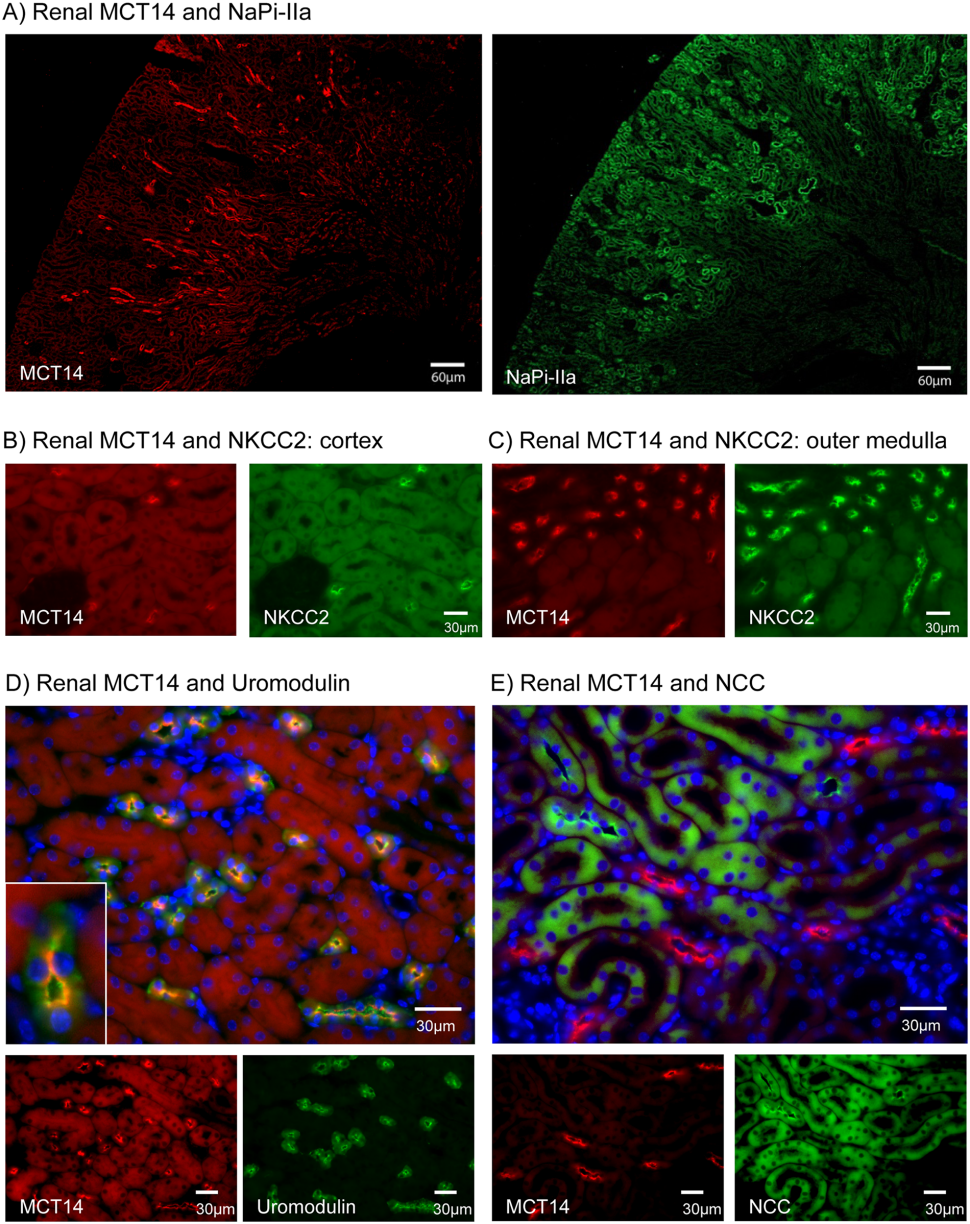
MCT14 renal localization. Consecutive sections showing MCT14 and NaPi-IIa localization (A). Cortical (B) and outer medullary (C) staining patterns of MCT14 and NKCC2 on consecutive sections. Costainings performed with MCT14 and uromodulin (D) or NCC (E) on renal sections. MCT14 is shown in red, NaPi-IIa, NKCC2, uromodulin and NCC are represented in green and blue indicates nuclear fluorescence (DAPI).

In order to further characterize the segmental distribution of MCT14 in the murine kidney, (co)immunostainings were performed in single or consecutive renal sections using the MCT14 antibody as well as antibodies against proteins with well-established patterns of renal expression. NaPi-IIa, the major Na^+^-dependent phosphate transporter in kidney, was used as a marker for proximal tubules as its expression is restricted to the apical membrane of this nephron segment [28]. Incubation of consecutive sections with antibodies against MCT14 and NaPi-IIa showed that both proteins labelled different nephron segments, with the MCT14 related signal mostly detected in the outer medulla whereas NaPi-IIa showed the expected cortical expression (Fig 3A), thus indicating that MCT14 is not expressed in proximal tubule. The NKCC2 cotransporter is expressed in the brush border membrane of the thick ascending limb of the loop of Henle (TAL), where it mediates the electroneutral reabsorption of 1Na^+^:1K^+^.2Cl^-^ [34]. Immunostainings on consecutive sections with antibodies against MCT14 and NKCC2 [25] showed that both proteins are present in the same cells, in cortex and outer medulla (Fig 3B and 3C). TAL expression of MCT14 was further supported by co-staining of MCT14 and uromodulin (Tamm-Horsfall protein), a GPI-anchored protein exclusively produced by the cells of the TAL and secreted into the urine upon proteolytic cleavage [35]. Upon coincubation of renal slices with antibodies against MCT14 and uromodulin, all cells positive for MCT14 stained also for uromodulin (Fig 3D). As expected, uromodulin staining was present intracellularly as well as on the luminal membrane of those cells, whereas MCT14 signal was restricted to the apical membrane. In order to investigate if MCT14 is exclusively expressed in the TAL or its expression extends into the adjacent distal convoluted tubule (DCT), further co-stainings were performed with a marker of the DCT, namely the Na^+^/Cl^-^ cotransporter NCC [36]. Coincubation with antibodies against MCT14 and NCC [27] resulted in labeling of different tubular segments (Fig 3E), thus confirming that MCT14 is located specifically in TAL.

### Dietary phosphate content regulates renal MCT14 protein abundance

**Fig 4:**
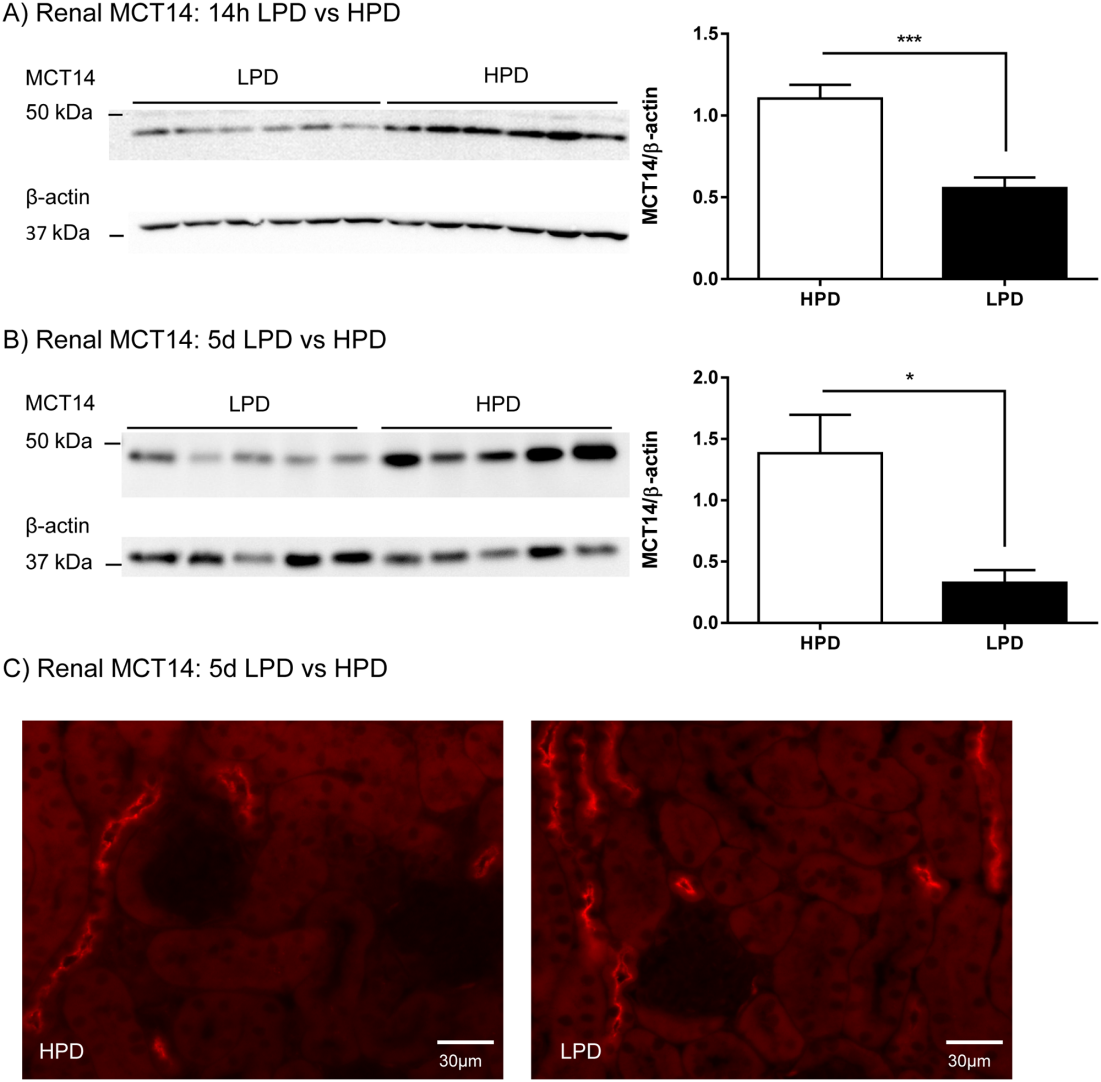
Regulation of MCT14 protein abundance by dietary phosphate. Western blotting with MCT14 antibody was performed on renal homogenates from animals fed 14 hours (A) and 5 days (B) with either low (LPD) or high (HPD) phosphate. Expression levels were normalized to corresponding β-actin levels. MCT14 protein abundance was significantly increased in animals fed a HPD compared to LPD as indicated in the corresponding densitometric analysis. Statistical differences were calculated with t-test: *p<0.05 and ***p<0.001. Immunofluorescence staining against MCT14 was performed in kidneys of mice fed HPD or LPD for 5 days (C).

Western blot analysis of renal homogenates from animals used previously for transcriptome analysis showed that MCT14 protein abundance followed the regulation of mRNA by dietary phosphate. Reduced amounts of MCT14 protein was detected after 14 hours and 5 days LPD compared to HPD (Fig 4A and 4B). Immunostainings on renal cryosections of mice chronically adapted to HPD and LPD did not show a change of expression pattern, with the cortical-medullary gradient remaining similar as the one already observed in the initial localization experiments (Fig 4C).

### Functional studies in *Xenopus laevis* oocytes

To search for transport substrates of MCT14, we used *Xenopus* oocytes injected with/without Slc16a14/MCT14 cRNA. As indicated above, Western blot analysis confirmed that the protein was produced by the oocytes (see Fig 2C). Trafficking of MCT14 to the membrane of the oocytes was ascertained by immunostaining experiments, since proper membrane expression is required for transport studies. Oocytes injected with Slc16a14/MCT14 cRNA showed a signal framing the cell body, which was absent from non-expressing cells (Fig 2F), indicating insertion of MCT14 into the oocyte cell membrane.

**Fig 5:**
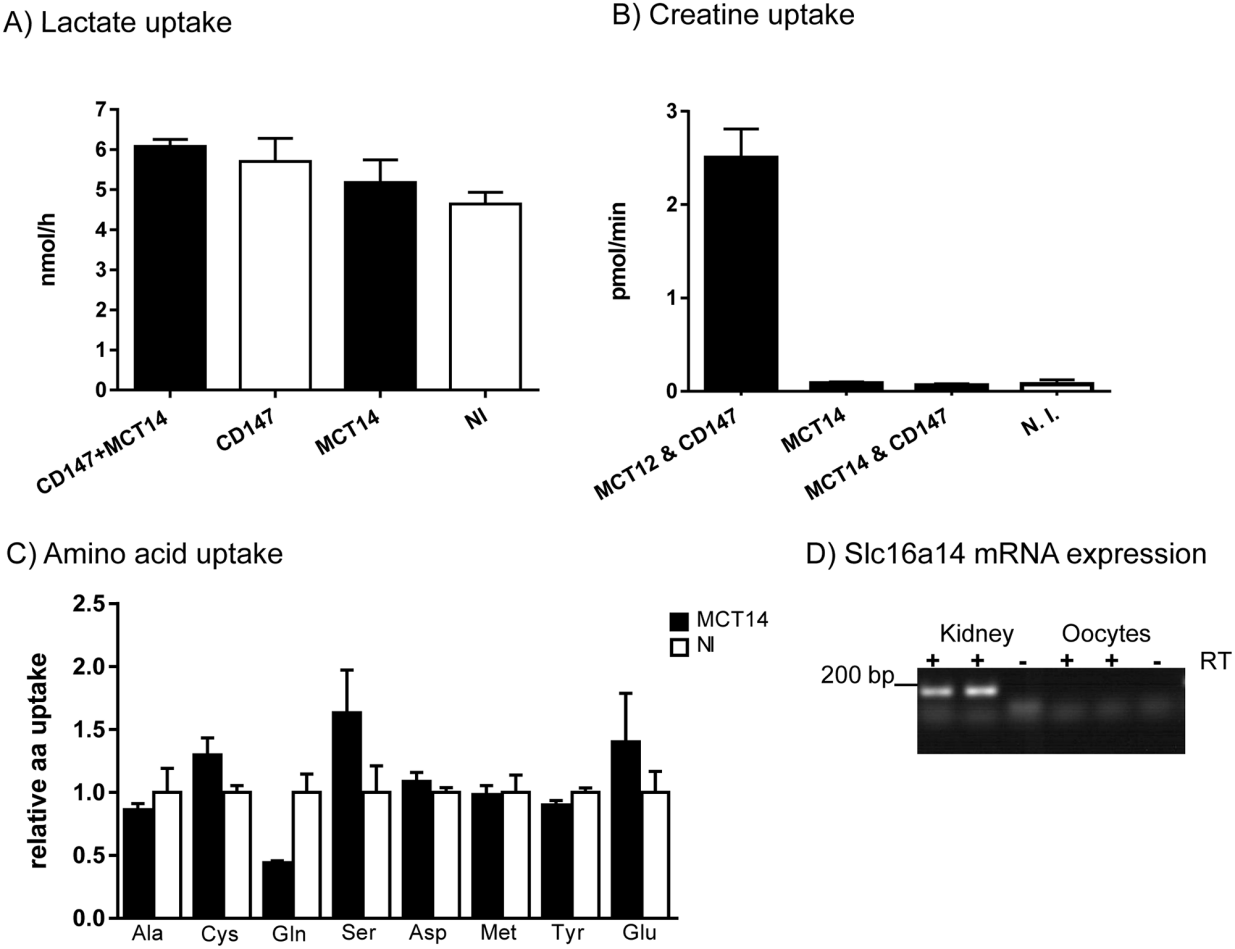
Uptake in *X*. *laevis* oocytes. Lactate transport was assessed in *Xenopus laevis* oocytes injected with cRNA of MCT14 and CD147, in oocytes injected with MCT14 or CD147 alone and non-injected oocytes (NI) (A). Oocytes expressing MCT12 and CD147, MCT14, MCT14 and CD147 and non-expressing oocytes (N.I.) were used to perform creatine uptake (B). Transport of the indicated amino acids into oocytes expressing MCT14 is given in relation of uptakes into non-injected oocytes (NI) (C). Slc16a14 mRNA was detected in *Xenopus laevis* kidney but not oocytes (D). Each experiment was performed with at least n = 7–10 oocytes.

Oocytes expressing MCT14 and non-injected oocytes were incubated with several substrates previously shown to be transported by different members of the MCT family. The physiological role of the classical monocarboxylate transporters MCT1-4 is the import and export of lactate as a substrate of oxidation or end product of glycolysis. MCT1, 3 and 4 interact with the accessory protein CD147 and for MCT1 it is well documented that association with CD147 is required for proper targeting to the basolateral membrane of the proximal tubule [11]. However, oocytes injected with Slc16a14/MCT14 mRNA did not show any significant transport activity for lactate compared to non-injected oocytes, neither at substrate concentrations of 3 mM (Fig 5A) or at 100 μM (Table 1). Coexpression of MCT14 with CD147 also failed to induce lactate transport. Previous experiments detected CD147 at the basolateral membrane of TAL cells [12] whereas MCT14 appears to be restricted to the apical membrane of these cells making a potential direct interaction between these two proteins in vivo rather unlikely. Phylogenetically MCT14 is closer related to MCT8 and MCT10, than to the classical monocarboxylate transporters. MCT8 and MCT10 transport thyroid hormone [14] and aromatic amino acids [37] respectively. A recent study suggested that MCT14 might be involved in transport of amino acids, rather than in the shuttling of monocarboxylates [24]. Therefore, further uptake experiments were performed in which oocytes were incubated with different amino acids (alanine, glutamine, cysteine, asparagine, serine, methionine, tyrosine and glutamate) in 100 μM concentrations. No significant differences were observed comparing oocytes expressing MCT14 and the corresponding non-expressing cells, except for a tendency of slightly higher import of serine and glutamate, which could be due to structural homologies to endogenous substrate (Fig 5C, Table 1). Similarly, MCT14 failed to induce transport of tryptophan, phenylalanine, valine, leucine and isoleucine, with substrates present at 3 mM concentrations (Table 1). However, the rate of induction was very small and may represent rather the stimulation of an endogenous *Xenopus* transport system than transport by MCT14 itself. Since most amino acids are already absorbed in the proximal tubule, very low concentrations are reaching the TAL; therefore, amino acid transporters located in the TAL would be expected to have rather the properties of a high affinity transporter. Another member of the MCT family, which has been recently characterized, is MCT12. MCT12 has been shown to be a creatine transporter [17]. However, creatine was not transported either by oocytes injected with MCT14, whereas this substrate was incorporated by MCT12 expressing oocytes (Fig 5B). Introduction of CD147 had again no effect in Slc16a14/MCT14 expressing oocytes regarding their failure to transport creatine. Similarly, inorganic phosphate was not accumulated in oocytes expressing MCT14 (Table 1). Of interest, Ansermet *et al* just reported a renal Xpr1 knock out mouse model, which develops hypophosphatemic rickets, and in which Slc16a14 mRNA was strongly suppressed [38], similar to our observations under low dietary phosphate content. Xpr1 is a protein thought to be involved in phosphate export [39], though its direct function remains to be proven. Although we failed to show phosphate transport activity, MCT14 might be involved in the metabolic adaptation to alterations in phosphate homeostasis. No endogenous Slc16a14 mRNA was detected in *Xenopus laevis* oocytes, whereas the same amount of mRNA from *Xenopus laevis* kidney resulted in a strong band of expected size (Fig 5D). This indicates that MCT14 is also expressed in kidney of *Xenopus laevis* but not in oocytes, where it could interfere with transport studies.

**Table 1:** Transport studies in *Xenopus laevis* oocytes [pmol/10min].

Substrate	MCT14	Non injected
Lactate	7.593±0.600	7.174±1.620
Pyruvate	0.300±0.063	1.177±0.169*
Alanine	4.430±0.702	5.135±2.803
Methionine	4.784±0.906	4.863±1.789
Cysteine	0.635±0.177	0.489±0.076
Cystine	6.601±1.419	7.866±0.962
Glutamine	2.676±0.274	6.053±2.502*
Serine	12.29±7.19	7.52±4.49
Tyrosine	11.44±1.05	12.69±1.32
Glutamate	0.882±0.685	0.628±0.278
Aspartate	1.770±0.327	1.625±0.176
Isoleucine	4.955±6.238	1.997±2.152
Valine	25.70±1.97	24.70±3.23
Tryptophane	90.97±14.58	100.93±8.41
Phenylalanine	81.18±23.12	86.28±15.39
Phosphate	1.384±0.479	1.376±0.377
Urate	0.0039±0.0015	0.0087±0.0039*
Citrate	6.442±3.022	5.452±0.462

In summary, we showed that the renal abundance of MCT14 is highly regulated by the dietary content of phosphate and that its expression in kidney is restricted to the luminal side of the TAL. However, MCT14 seems to transport neither phosphate nor several other substrates of different members of the MCT family.
